# Supplementary material for: Heterophilic interactions between cell-surface proteins Teneurin-m and Capricious drive dendrite segregation in the Drosophila olfactory circuit
Source: bioRxiv. 2026 Jul 14:2026.04.22.719985. Originally published 2026 Apr 27. Preprint. [Version 2] doi: 10.64898/2026.04.22.719985 (PMC13148107; doi:10.64898/2026.04.22.719985)
Supplement: 1 [file NIHPP2026.04.22.719985V2-supplement-1.pdf]

bioRxiv preprint doi: https://doi.org/10.64898/2026.04.22.719985; this version posted July 14, 2026. The copyright holder for this preprint (which was not certified by peer review) is the author/funder, who has granted bioRxiv a license to display the preprint in perpetuity. It is made available under a CC-BY 4.0 International license.

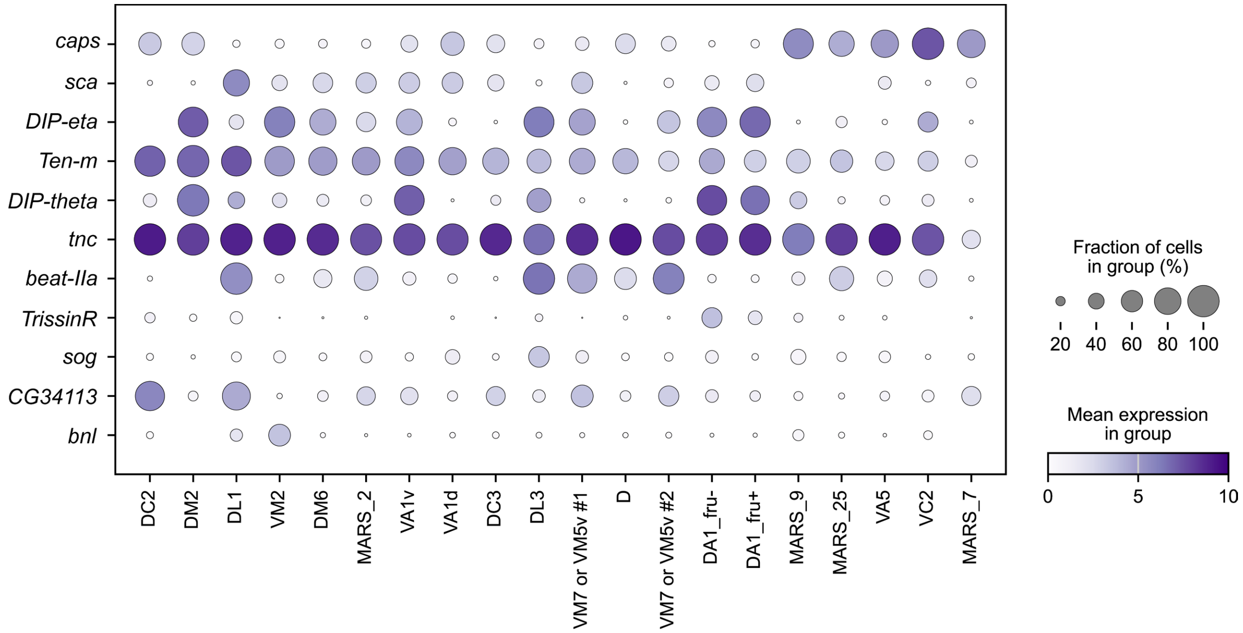

Figure S1. Top 10 candidate cell-surface protein genes with expression anti-correlated to *caps* in projection neurons at 12 h APF, related to Figure 1 and Figure 2.

Expression profiles of *caps* and top 10 anti-correlated genes at 12 h APF, shown across PN types. Dot size, percentage of expressing cells; color intensity, mean expression level [log_2(CPM+1), where CPM is counts per million reads]. The *caps* and *Ten-m* rows are reproduced as Figure 3A.

bioRxiv preprint doi: https://doi.org/10.64898/2026.04.22.719985; this version posted July 14, 2026. The copyright holder for this preprint (which was not certified by peer review) is the author/funder, who has granted bioRxiv a license to display the preprint in perpetuity. It is made available under a CC-BY 4.0 International license.

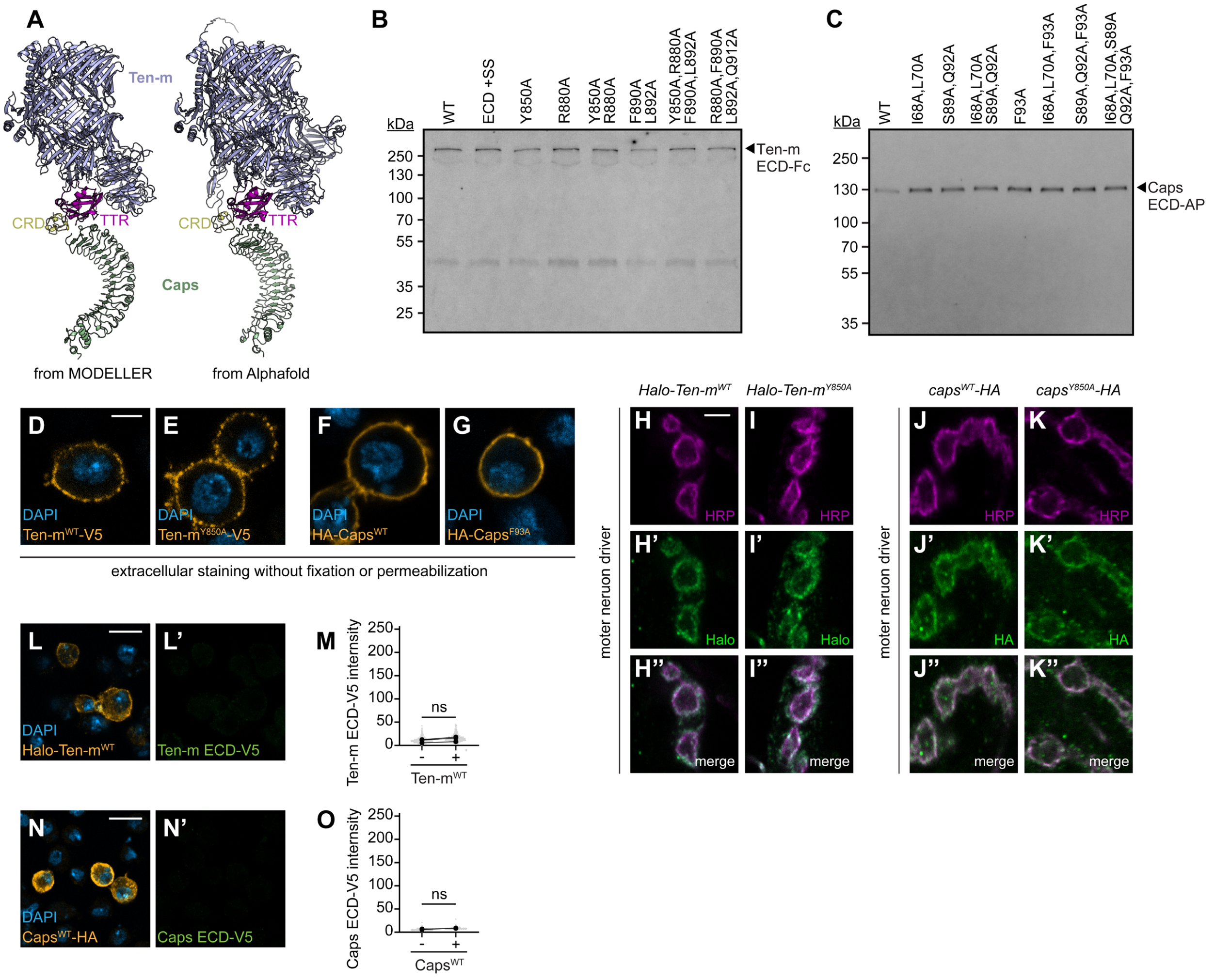

Figure S2. Expression and subcellular localization of Ten-m and Caps variants, related to Figure 2.

(A) Model of Ten-m–Caps complex, derived via homolog modeling from our experimentally determined TEN-1–LRON-11 structure (left), closely resembles the AlphaFold2-derived Ten-m–Caps model (right). Low confidence (low pLDDT, predicted local distance difference test) residues were hidden in the AlphaFold2 model.

(B, C) Western blots of media of cultured cells expressing ECDs of Ten-m and Caps show that Ten-m and Caps mutant ECDs express and secrete, which are then used in the ECIA. Molecules were detected using an anti-pentaHis antibody that recognizes both Fc- and AP-tagged constructs via a common C-terminal hexahistidine tag.

(D–G) Surface expression of Ten-m and Caps variants in S2 cells. Live staining of extracellular epitopes confirms proper membrane trafficking: Ten-m^{WT}-V5 (D), Ten-m^{Y850A}-V5 (E), HA-Caps^{WT} (F), or HA-Caps^{F93A} (G). Scale bar, 3 µm.

(H–K”) Neuromuscular junction localization of intracellularly tagged constructs expressed via *D42-GAL4* expressed in motor neurons. All variants (labeled on top) colocalize with neuronal membranes at HRP-positive synaptic boutons at the neuromuscular junction, confirming protein stability and axonal transport *in vivo*. Scale bar, 3 µm.

bioRxiv preprint doi: https://doi.org/10.64898/2026.04.22.719985; this version posted July 14, 2026. The copyright holder for this preprint (which was not certified by peer review) is the author/funder, who has granted bioRxiv a license to display the preprint in perpetuity. It is made available under a CC-BY 4.0 International license.

(L–L’, N–N’) Cell-based binding assays testing homophilic interactions. S2 cells expressing Halo-Ten-m^{WT} incubated with soluble Ten-m ECD-V5 (L–L’) and S2 cells expressing $Caps^{WT}$-HA incubated with soluble Caps ECD-V5 (N–N’). Scale bars, 10 μm.

(M, O) Quantification of Ten-m ECD-V5 (M) and Caps ECD-V5 (O) binding intensity on cells expressing indicated receptors versus untransfected controls. Paired *t*-test (ns, not significant). Gray dots, individual cell intensities; black dots, experimental means (n = 3, 70–909 cells per experiment). Lines connect paired untransfected and transfected populations from the same slide.

bioRxiv preprint doi: https://doi.org/10.64898/2026.04.22.719985; this version posted July 14, 2026. The copyright holder for this preprint (which was not certified by peer review) is the author/funder, who has granted bioRxiv a license to display the preprint in perpetuity. It is made available under a CC-BY 4.0 International license.

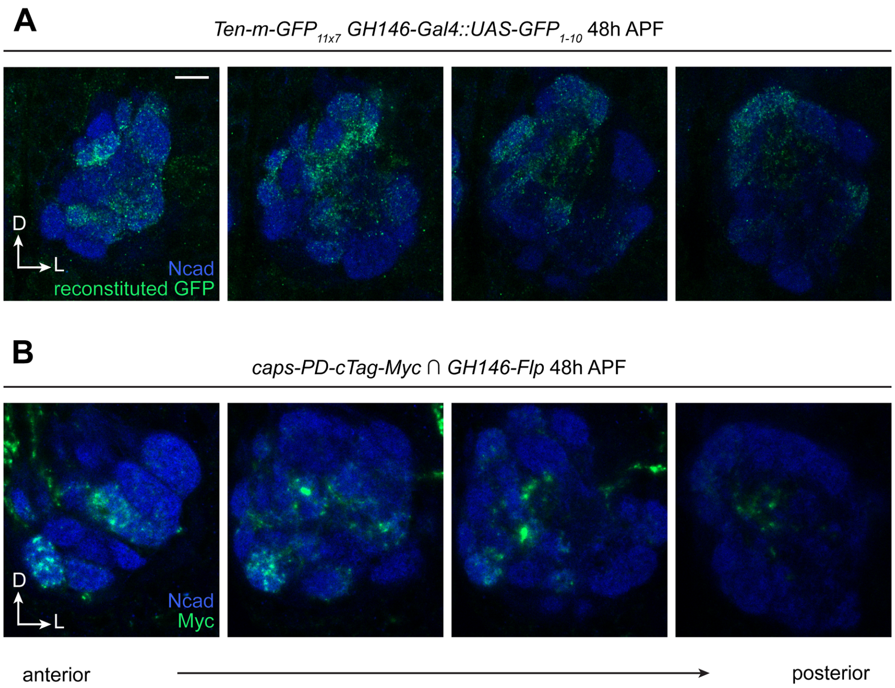

Figure S3. Endogenous Ten-m and Caps expression patterns in PNs at 48 h APF, related to Figure 3.

(A) Split-GFP visualization of endogenous Ten-m proteins in PNs. Ten-m is endogenously tagged with *GFP*$_{11\times7}$[27]; *GFP*$_{1\text{–}10}$ is expressed under *GH146-GAL4* driver. Reconstituted GFP detected by immunostaining with a conformation-specific anti-GFP antibody.

(B) Conditional tagging strategy for PN-specific Caps visualization using *Caps-PD-HA>stop>Myc* intersected with *GH146-Flp*, detected via anti-Myc immunostaining.

Both panels show a representative antennal lobe in four single confocal sections from anterior to posterior. Scale bars, 10 µm. Note that *GH146* drivers are expressed in most but not all PN types, limiting detection coverage.

bioRxiv preprint doi: https://doi.org/10.64898/2026.04.22.719985; this version posted July 14, 2026. The copyright holder for this preprint (which was not certified by peer review) is the author/funder, who has granted bioRxiv a license to display the preprint in perpetuity. It is made available under a CC-BY 4.0 International license.

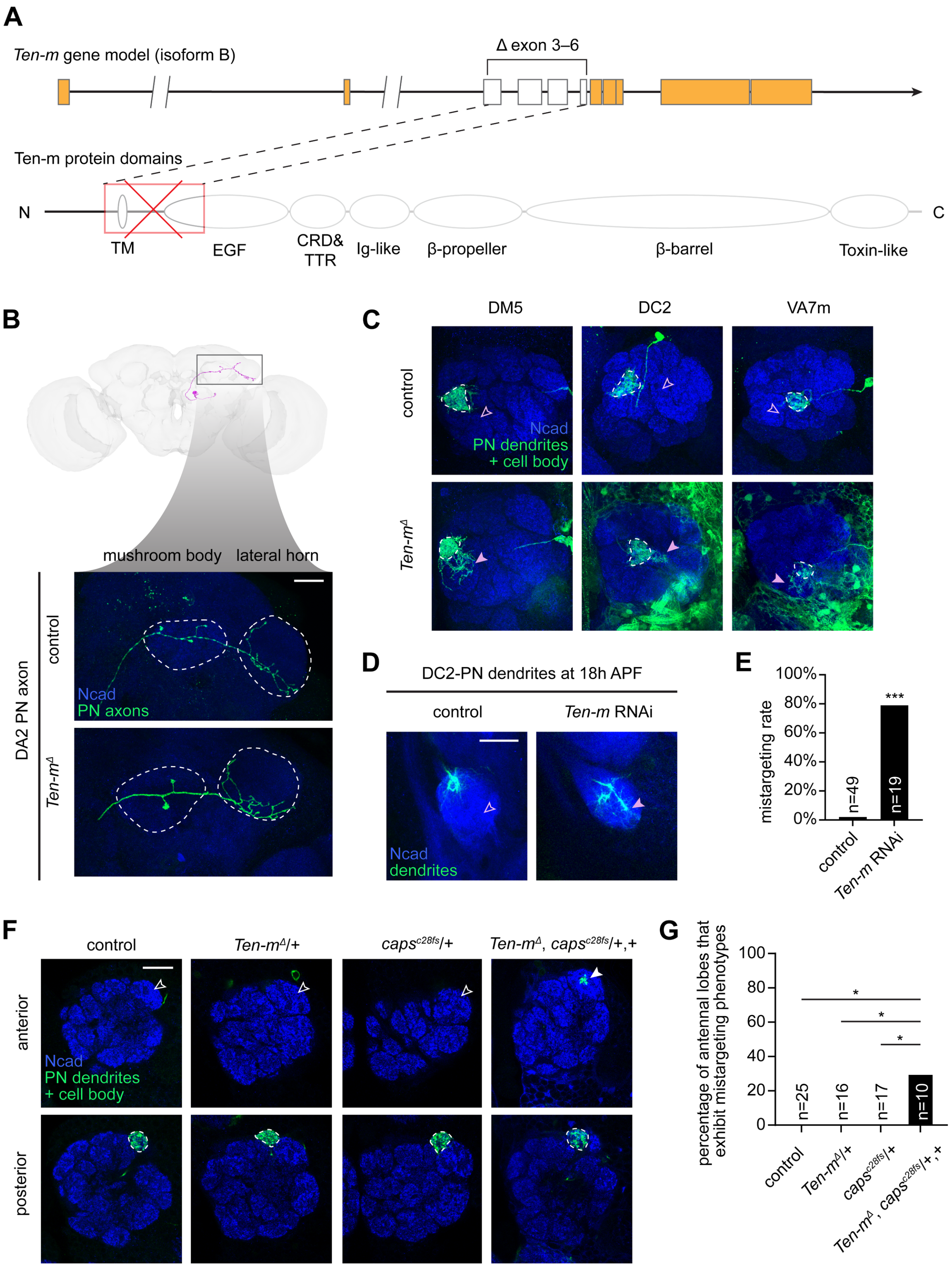

Figure S4. *In vivo* characterization of *Ten-m* loss-of-function and its genetic interaction with *caps*, related to Figure 4.

(A) Top: Schematic of the *Ten-m*$^{\Delta}$ allele showing the gene model of isoform B (filled boxes, coding exons; lines, introns; exons 3–6 shown as open boxes within the bracket-marked deletion). Double slashes

bioRxiv preprint doi: https://doi.org/10.64898/2026.04.22.719985; this version posted July 14, 2026. The copyright holder for this preprint (which was not certified by peer review) is the author/funder, who has granted bioRxiv a license to display the preprint in perpetuity. It is made available under a CC-BY 4.0 International license.

(//) indicate a large intron drawn not to scale. Bottom: Ten-m protein domain architecture (isoform B) with major domains depicted as labeled shapes. Dashed lines connect the deleted exons to the TM-encoding region of the protein. The deletion eliminates the TM domain (red cross) and the resulting frameshift truncates all downstream extracellular sequence (shown faded), generating a predicted null protein product. The deletion is predicted to disrupt all Ten-m isoforms.
(B) Tracings of a DA2-PN from the FlyWire dataset[31–33]; boxed region indicates axon branching in the mushroom body and lateral horn (top). Representative confocal images of DA2-PN axon morphology in control and $Ten\text{-}m^{\Delta}$ mutants. Mutant clones were unambiguously identified as DA2-PNs based on their stereotyped axonal arborization in the lateral horn. This morphology is clearly distinct from that of DM5-PNs, the other principal lateral PN generated during the same 96–120 h heat-shock window.
(C) Examples of dendrite mistargeting in $Ten\text{-}m^{\Delta}$ PNs. Dashed lines, native glomerular targets; open/filled pink arrowheads, normally uninnervated/ectopically innervated regions.
(D) DC2-PN dendrite mistargeting upon *Ten-m* knockdown at 18–21 h APF. Dorsal-to-ventral shift indicated by empty/filled pink arrows.
(E) Quantification of DC2-PN mistargeting. 2×2 Fisher's Exact Test; ***, $p < 0.001$.
(F) DL3-PN dendrite mistargeting in DA1 glomerulus in *Ten-m*/*caps* trans-heterozygotes, but not in wild-type controls or single-heterozygotes. Dashed lines outline DL3 glomerulus; open/filled arrowheads, normally uninnervated/ectopically innervated regions.
(G) Quantification of percentage of antennal lobes that exhibit mistargeting phenotypes across genotypes. Statistical differences between conditions were assessed using 2×2 Fisher's Exact Test followed by Benjamini-Hochberg False Discovery Rate (FDR) correction for 3 pre-planned comparisons (trans-heterozygotes vs. control and each single heterozygote). *, adjusted $p < 0.05$.

bioRxiv preprint doi: https://doi.org/10.64898/2026.04.22.719985; this version posted July 14, 2026. The copyright holder for this preprint (which was not certified by peer review) is the author/funder, who has granted bioRxiv a license to display the preprint in perpetuity. It is made available under a CC-BY 4.0 International license.

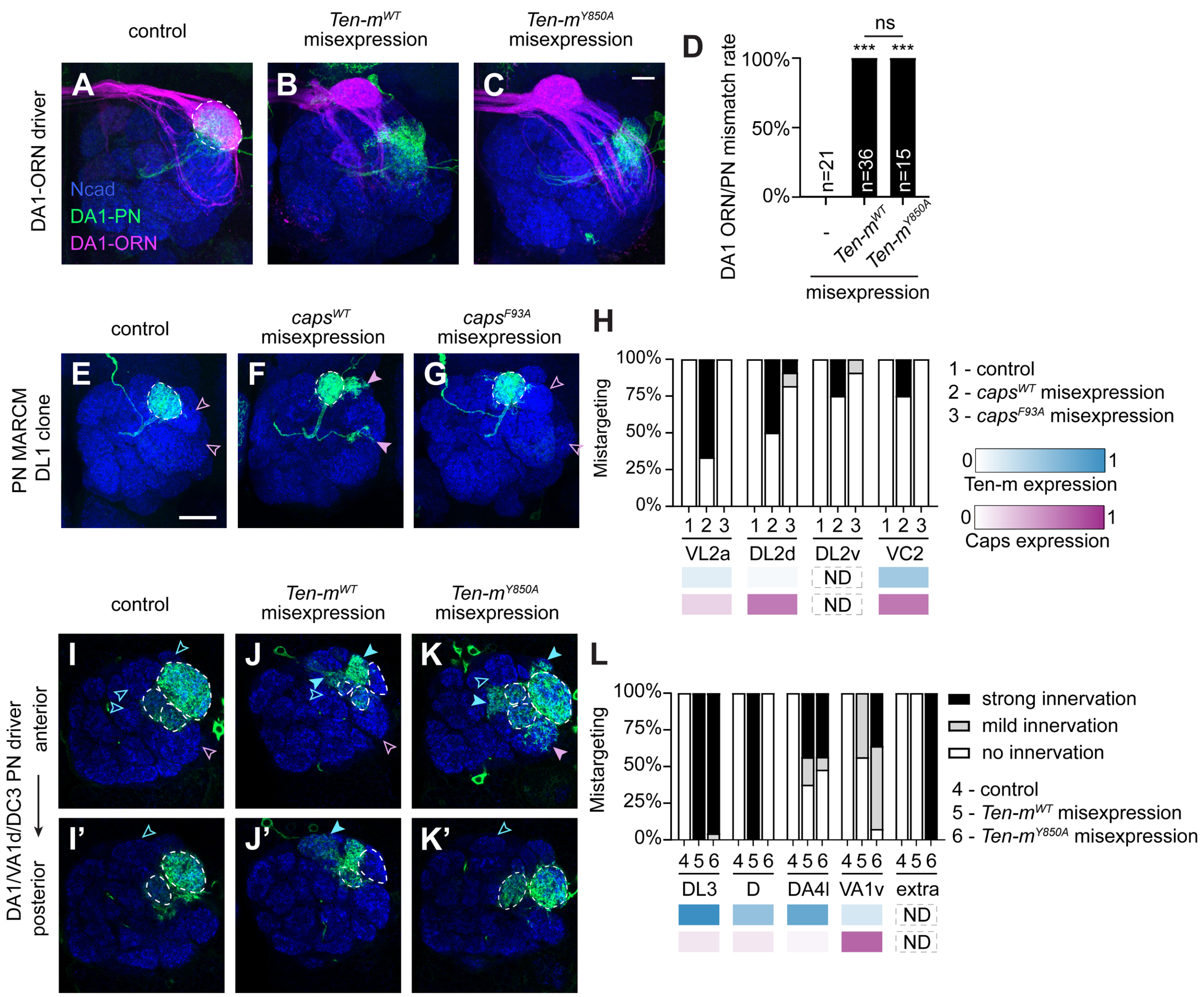

Figure S5. Gain-of-function analysis of Ten-m and Caps in ORN–PN matching and dendrite segregation, related to Figure 5.

(A–C) Confocal images showing DA1-ORN axons and cognate DA1-PNs for control (A), *Ten-m*^{WT}-misexpression in DA1-ORNs (B), and *Ten-m*^{Y850A}-misexpression in DA1-ORNs (C). Instead of matching with DA1-PNs, misexpression of both *Ten-m*^{WT} and *Ten-m*^{Y850A} causes DA1-ORNs to mistarget to the DL3 glomeruli[13]. Scale bar, 10 µm.

(D) Quantification of mistargeting penetrance. Statistical differences between conditions were assessed using 2×2 Fisher's Exact Test followed by Benjamini-Hochberg (FDR) correction for all pairwise comparisons. ns (not significant); ***, $p < 0.001$.

(E–G) Confocal images showing DL1-PN MARCM clones for control (E), *caps*^{WT} misexpression (F), and *caps*^{F93A} misexpression (G). Open/filled pink arrowheads indicate normally uninnervated and ectopically innervated Ten-m-low regions, respectively. Misexpression of *caps*^{WT} causes DL1-PN dendrites to invade Ten-m-low regions, whereas *caps*^{F93A} misexpression shows minimal effect. Scale bar: 20 µm.

(H) Quantification of DL1-PN mistargeting penetrance for genotypes shown in E–G, with corresponding protein expression profiles at 48 h APF (n = 9, 12, 11 antennal lobes, respectively).

bioRxiv preprint doi: https://doi.org/10.64898/2026.04.22.719985; this version posted July 14, 2026. The copyright holder for this preprint (which was not certified by peer review) is the author/funder, who has granted bioRxiv a license to display the preprint in perpetuity. It is made available under a CC-BY 4.0 International license.

(I–K') Confocal images showing Mz19-PN dendrites (*Mz19-GAL4*) for control (I, I'), *Ten-m*^{WT} misexpression (J, J'), and *Ten-m*^{Y850A} misexpression (K, K'). Open/filled arrowheads indicate normally uninnervated and ectopically innervated Caps-low (blue) or Caps-high (pink) regions, respectively. Control Mz19-PN dendrites target DA1, VA1d, DC3 glomeruli. *Ten-m*^{WT} misexpression causes Mz19 dendrites to mistarget to Caps-low DL3, D, DA4l regions (filled blue arrowheads in J, J'). *Ten-m*^{Y850A} misexpression causes mistargeting to Caps-low DL3, the formation of an extra, ectopic glomerulus (not present in control brains; filled blue arrowheads in K), as well as Caps-high VA1v (filled pink arrowhead in K).
(L) Quantification of Mz19-PN mistargeting penetrance for genotypes shown in I–K', with corresponding protein expression profiles at 48 h APF (n = 20, 16, 23 antennal lobes, respectively).

bioRxiv preprint doi: https://doi.org/10.64898/2026.04.22.719985; this version posted July 14, 2026. The copyright holder for this preprint (which was not certified by peer review) is the author/funder, who has granted bioRxiv a license to display the preprint in perpetuity. It is made available under a CC-BY 4.0 International license.

**Figure**	**Genotype**		
	**X**	**II**	**III**
**3C, 3D**			*caps-PD-HA>stop>Myc / V5-Ten-m*
**3E, S3A**		*GH146-GAL4 / +*	*Ten-m-GFP*$_{11x7}$ */ UAS-GFP*$_{1-10}$
**3F, S3B**		*GH146-Flp / +*	*caps-PD-HA>stop>Myc / +*
**4A', 4C, 4E', 4G, S4B, S4C, 5A', 5E', 5E", 5I, S5E, S5H (control)**	*UAS-mCD8-GFP, hsFlp*^{122}	*GH146-GAL4, UAS-mCD8-GFP / +*	*TubP-GAL80, FRT2A / FRT2A*
**4B', 4C, 4D, S4B, S4C, 5B', 5D (*****Ten-m*$^{\Delta}$**)**	*UAS-mCD8-GFP, hsFlp*^{122}	*GH146-GAL4 UAS-mCD8-GFP / +*	*TubP-GAL80, FRT2A / Ten-m*$^{\Delta}$*, FRT2A*
**4F', 4G, 4H, 5F', 5F" (*****caps*^{c28fs}**)**	*UAS-mCD8-GFP, hsFlp*^{122}	*GH146-GAL4, UAS-mCD8-GFP / +*	*TubP-GAL80, FRT2A / caps*^{c28fs}*, FRT2A*
**S4D, S4E (control)**		*GH146-Flp, UAS>>CAAX-Halo / +*	*91G04-GAL4 / +*
**S4D, S4E (*****Ten-m*** **RNAi)**	*UAS-dcr2*	*GH146-Flp, UAS>>CAAX-Halo / +*	*91G04-GAL4 / UAS-Ten-m-RNAi*
**S4F, S4G (control)**		*GMR37H08-LexA, LexAop-tdTomato*	*+ / +*
**S4F, S4G (*****Ten-m*** $^{\Delta}$ / +**)**		*GMR37H08-LexA, LexAop-tdTomato*	*Ten-m* $^{\Delta}$ */ +*
**S4F, S4G (*****caps*^{c28fs} / +**)**		*GMR37H08-LexA, LexAop-tdTomato*	*caps*^{c28fs} */ +*
**S4F, S4G (*****Ten-m*** $^{\Delta}$**,** ***caps*^{c28fs} / +, +**)**		*GMR37H08-LexA, LexAop-tdTomato*	*Ten-m* $^{\Delta}$ */ caps*^{c28f}
**5C', 5D (*****Ten-m*** ^{Y850A}**)**	*UAS-mCD8-GFP, hsFlp*^{122}	*GH146-GAL4, UAS-mCD8-GFP / +*	*TubP-GAL80, FRT2A / Ten-m*^{Y850A}*, FRT2A*
**5G', 5G", 5I (*****caps*^{c28fs}**,** *caps*^{WT} **rescue)**	*UAS-mCD8-GFP, hsFlp*^{122}	*GH146-GAL4, UAS-mCD8-GFP / UAS-caps*^{WT}	*TubP-GAL80, FRT2A / caps*^{c28fs}*, FRT2A*
**5H', 5H", 5I (*****caps*^{c28fs}**,** *caps*^{F93A} **rescue)**	*UAS-mCD8-GFP, hsFlp*^{122}	*GH146-GAL4, UAS-mCD8-GFP / UAS-caps*^{F93A}	*TubP-GAL80, FRT2A / caps*^{c28fs}*, FRT2A*
**S5A, S5D (control)**	*UAS-dcr2, UAS-CD8-GFP / +*	*Mz19-QF2G4HACK, QUAS-mtdTomato-3xHA, VT028327-p65*^{AD} */ +*	*GMR22E04-GAL4*^{DBD} */ +*
**S5B, S5D (*****Ten-m*^{WT} **misexpression)**	*UAS-dcr2, UAS-CD8-GFP / +*	*Mz19-QF2G4HACK, QUAS-mtdTomato-3xHA, VT028327-p65*^{AD} */ UAS-Ten-m*^{WT}	*GMR22E04-GAL4*^{DBD} */ +*
**S5C, S5D (*****Ten-m*^{Y850A} **misexpression)**	*UAS-dcr2, UAS-CD8-GFP / +*	*Mz19-QF2G4HACK, QUAS-mtdTomato-3xHA, VT028327-p65*^{AD} */ UAS-Ten-m*^{Y850A}	*GMR22E04-GAL4*^{DBD} */ +*
**S5F, S5H (*****caps*^{WT} **misexpression)**	*UAS-mCD8-GFP, hsFlp*^{122}	*GH146-GAL4, UAS-mCD8-GFP / UAS-caps*^{WT}	*TubP-GAL80, FRT2A / FRT2A*
**S5G, S5H (*****caps*^{F93A} **misexpression)**	*UAS-mCD8-GFP, hsFlp*^{122}	*GH146-GAL4, UAS-mCD8-GFP / UAS-caps*^{F93A}	*TubP-GAL80, FRT2A / FRT2A*
**S5I, S5I', S5L (control)**		*MZ19-GAL4, UAS-mCD8-GFP / +*	

bioRxiv preprint doi: https://doi.org/10.64898/2026.04.22.719985; this version posted July 14, 2026. The copyright holder for this preprint (which was not certified by peer review) is the author/funder, who has granted bioRxiv a license to display the preprint in perpetuity. It is made available under a CC-BY 4.0 International license.

**S5J, S5J', S5L (*Ten-m*^{WT} misexpression)**		*MZ19-GAL4, UAS-mCD8-GFP / UAS-Ten-m*^{WT}	
**S5K, S5K', S5L (*Ten-m*^{Y850A} misexpression)**		*MZ19-GAL4, UAS-mCD8-GFP / UAS-Ten-m*^{Y850A}	

Table S1. Complete genotypes of each experiment, related to STAR Methods.
